# Different Angiogenic Potentials of Mesenchymal Stem Cells Derived from Umbilical Artery, Umbilical Vein, and Wharton's Jelly

**DOI:** 10.1155/2017/3175748

**Published:** 2017-08-10

**Authors:** Lu Xu, Jianjun Zhou, Jingyu Liu, Yong Liu, Lei Wang, Ruiwei Jiang, Zhenyu Diao, Guijun Yan, Bruno Pèault, Haixiang Sun, Lijun Ding

**Affiliations:** ^1^Center for Reproductive Medicine, Department of Obstetrics and Gynecology, The Affiliated Drum Tower Hospital of Nanjing University Medical School, 321 Zhongshan Rd., Nanjing 210008, China; ^2^Central Research Lab, The Affiliated Drum Tower Hospital of Nanjing University Medical School, 321 Zhongshan Rd., Nanjing 210008, China; ^3^MRC Center for Regenerative Medicine, University of Edinburgh, Edinburgh EH16 4UU, UK; ^4^Orthopedic Hospital Research Center and Broad Stem Cell Center, David Geffen School of Medicine, University of California, Los Angeles, CA, USA; ^5^Clinical Center for Stem Cells, The Affiliated Drum Tower Hospital of Nanjing University Medical School, 321 Zhongshan Rd., Nanjing 210008, China

## Abstract

Human mesenchymal stem cells derived from the umbilical cord (UC) are a favorable source for allogeneic cell therapy. Here, we successfully isolated the stem cells derived from three different compartments of the human UC, including perivascular stem cells derived from umbilical arteries (UCA-PSCs), perivascular stem cells derived from umbilical vein (UCV-PSCs), and mesenchymal stem cells derived from Wharton's jelly (WJ-MSCs). These cells had the similar phenotype and differentiation potential toward adipocytes, osteoblasts, and neuron-like cells. However, UCA-PSCs and UCV-PSCs had more CD146^+^ cells than WJ-MSCs (*P* < 0.05). Tube formation assay in vitro showed the largest number of tube-like structures and branch points in UCA-PSCs among the three stem cells. Additionally, the total tube length in UCA-PSCs and UCV-PSCs was significantly longer than in WJ-MSCs (*P* < 0.01). Microarray, qRT-PCR, and Western blot analysis showed that UCA-PSCs had the highest expression of the Notch ligand Jagged1 (JAG1), which is crucial for blood vessel maturation. Knockdown of Jagged1 significantly impaired the angiogenesis in UCA-PSCs. In summary, UCA-PSCs are promising cell populations for clinical use in ischemic diseases.

## 1. Introduction

Over the last few decades, mesenchymal stem cells (MSCs) have been widely explored for their potential as a treatment strategy for disorders caused by insufficient angiogenesis, including atherosclerosis, stroke, myocardial infarction, and chronic wounds [1]. These cells have several characteristic features. First, they can adhere to tissue culture flasks and are positive for specific markers like CD73, CD90, and CD105 and negative for hematopoietic markers such as CD34, CD45, and HLA-DR. Second, they can differentiate into adipocytes, osteoblasts, and chondrocytes in vitro [2]. MSCs can be isolated from many human tissues such as bone marrow, adipose tissue, peripheral blood, dental pulp, placenta, amniotic fluid, umbilical cord (UC), pancreas, and spleen [3–5]. In recent years, UC has been acknowledged to be a better source of MSCs. Besides the noninvasive collection procedure, no ethical issues, and faster self-renewal, UC-derived MSCs have been shown to be multipotent and immunomodulatory [6, 7]. Currently, UC-derived MSCs are isolated primarily from Wharton's jelly (WJ-MSCs), which is the mucoid connective tissue in the UC [8]. Actually, there are three large vessels surrounded by the WJ, which is enveloped in the amniotic epithelium, including two umbilical arteries (UCAs) and one umbilical vein (UCV). Previous reports have found that human UC perivascular cells, including UCA perivascular stem cells (UCA-PSCs) and UCV perivascular stem cells (UCV-PSCs), are distinctly different from WJ-MSCs [9]. In particular, CD146^+^ UC perivascular cells have been found to express typical MSCs markers and could accelerate wound healing by enhancing angiogenesis [10, 11].

MSCs mainly originate from two types of perivascular cells, pericytes (CD45^−^CD31^−^CD146^+^CD34^−^) and adventitial cells (CD45^−^CD31^−^CD146^−^CD34^+^), which contain the in situ counterpart of MSCs in human organs and yield a progeny of multilineage mesodermal progenitor cells [12, 13]. Recently, osteogenic and adipogenic progenitors have also been shown to originate from perivascular niches in vivo and purified pericytes [14–16]. In addition, transplantation of purified pericytes can support vasculature and repair damaged tissue [17, 18]. These results indicate the therapeutic capacity of perivascular stem cells in postinjury angiogenesis/vasculogenesis.

Although many previous studies have identified cell populations arising from specific cord regions, it remains to be unknown if UCA-PSCs, UCV-PSCs, and WJ-MSCs from the same UC differ in terms of proliferation ability, differentiation ability, and especially angiogenic capacity [19–21]. Therefore, we described the basic characterization of UCA-PSCs, UCV-PSCs, and WJ-MSCs derived from the same UC and compared their angiogenic potential in vitro which may provide a new alternative source for cell-based therapeutic applications in ischemia.

## 2. Materials and Methods

### 2.1. Preparation of Human UC Sample

Human UC tissue samples (*n* = 10) were collected from the Affiliated Drum Tower Hospital of Nanjing University Medical School and processed within 12 h of natural delivery. The physician obtained verbal informed consent from the healthy mother without any pregnancy complication for the use of the umbilical cord in the present research. The experimental procedure was approved by the Clinical Research Ethics Committee at the Affiliated Drum Tower Hospital of Nanjing University Medical School. The UCs were then immersed in sterile phosphate-buffered saline (PBS, Gibco, Grand Island, NY, USA) supplemented with 5% penicillin/streptomycin (Gibco) for further tissue analysis or cell isolation.

### 2.2. Immunofluorescence Assay

UCA, UCV, and WJ were immersed in optimum cutting temperature (OCT) compound (Leica, Wetzlar, Germany) and frozen at −70°C until sectioning. The tissues were serially sectioned to 6 *μ*m thickness using a cryostat (Leica). Expression of PDGF-R*β* (ab32570, Abcam, Cambridge, UK), NG2 (ab139406, Abcam), *α*-SMA (ab5694, Abcam), and CD146 (ab75769, Abcam) was detected by immunofluorescence staining. After incubated with primary antibody at 4°C overnight, the frozen sections were then incubated with Alexa Fluor 488-conjugated goat anti-rabbit IgG (1 : 200, Invitrogen, Grand Island, NY, USA) or Alexa Fluor 555-conjugated goat anti-rabbit IgG (1 : 200, Invitrogen). The nuclei were stained with 4′,6-diamidino-2-phenylindole (DAPI), which was contained in the Vectashield mounting medium for fluorescence (Vector Laboratories Inc.). The images were visualized using fluorescence confocal microscopy (Leica) under a magnification of 600x. The integrated optical density (IOD) values of positive staining in five randomly selected fields of view were tested by Image pro-plus 6.0 software (Media Cybernetic, Rockville, USA).

### 2.3. Isolation and Culture of UCA-PSCs, UCV-PSCs, and WJ-MSCs

Adherent cells were isolated and cultured using the explant method. Briefly, two UCAs and one UCV were longitudinally extracted from human UC. The UCA, UCV, and WJ were then manually minced into 1-2 mm^3^ fragments. The vessels were cut in the direction perpendicular to the long axis with a sterile scissor. These fragments were aligned and seeded regularly on the tissue culture-treated dishes. As to the fragments minced from vessels, only the outlayer but not the cross section could touch the dish. Then, the culture medium containing low-glucose DMEM (LG-DMEM; Gibco) supplemented with 10% fetal bovine serum (FBS; Gibco), 1% penicillin/streptomycin (Gibco) and 10 ng/ml basic fibroblast growth factor (FGF2, Gibco) poured slowly and gently, cultured at 37°C and 5% CO_2_. The culture medium was replaced every 3–5 d for 2 weeks until fibroblast-like adherent cells reach 80–90% confluence. Then, adherent cells and tissue fragments were rinsed once with PBS and detached using a 0.05% trypsin/EDTA solution (TE; Gibco). The three types of stem cells were subpassaged at every 4-5 d with the ratio 1 : 4.

### 2.4. Proliferation Assay

Cell-counting kit-8 (CCK-8) (Dojindo, Kumarmoto, Japan) was used to measure the cell proliferation. Cells were seeded at 2 × 10^3^ cells per well into 96-cell plates, eight parallel wells for each group, which were conventionally cultured in complete medium (100 *μ*l/well) for every day in one week, respectively. The CCK-8 reagent (10 *μ*l) was added into each well. After incubation for 2 h, the optical density value (OD value) was measured at 450 nm position on a microplate reader (Thermo, Massachusetts, USA). Each well of cells was counted once. The growth curve was draw based on the mean value of the eight counts in each group. The culture medium was taken as blank control.

### 2.5. Flow Cytometry Analysis

The specific cell surface antigens of cultured cells (passage 3) were analyzed by flow cytometry using a FACScan flow cytometer (Becton Dickinson, USA). Single-cell suspensions were harvested from MSC cultures with 0.05% trypsin/EDTA (Gibco) and resuspended in PBS. The cells were filtered through a 70 *μ*m filter and incubated for 1 h with fluorescein isothiocyanate- (FITC-) or phycoreythrin- (PE-) conjugated antibodies against human CD13 (eBioscience, Colorado, USA), CD29 (eBioscience), CD34 (BD Pharmingen, San Diego, CA, USA), CD45 (eBioscience), CD73 (BD Pharmingen), CD90 (eBioscience), CD105 (eBioscience), CD146 (BD Pharmingen), and HLA-DR (eBioscience).

### 2.6. Multilineage Differentiation Assay

The cells at passage 3 were assessed for multipotency by adipogenic, osteogenic, and neural-like differentiation assays. Cells were seeded at a density of 5 × 10^3^/cm^2^ in 24-well plates and grown in monolayer in DMEM low glucose and FBS (10%) until reaching ~90% confluency, and then the cells were given the appropriate differentiation medium.

#### 2.6.1. Adipogenesis

Cells were cultured in adipogenic induction medium (Gibco). On day 14, cultures were stained with oil red O staining (Sigma) as an indicator of intracellular lipid accumulation.

#### 2.6.2. Osteogenesis

Cells were grown in osteogenic induction medium (Gibco) for 21 days. Calcium deposition was shown by alizarin red staining (Sigma, Steinheim, Germany).

#### 2.6.3. Neurogenesis

Cells were treated with the preinduction medium containing 10^−7^ mol/L all-trans-retinoic acid (ATRA, Sigma) and 10 ng/ml FGF2 (Gibco) for 18 h, and then with modified neuronal medium (MNM) for 36 h. The expression of neurofilament medium polypeptide (1 : 100, sc-16143, Santa Cruz Biotechnology, Santa Cruz, CA, USA) and neuron-specific anolase (1 : 100, sc-292097, Santa Cruz Biotechnology) in induced MSCs was detected by immunofluorescence staining.

### 2.7. Tube Formation Assay

Liquid Matrigel (BD Biosciences, USA, BD Matrigel Matrix Cat. No. 356234) was added into 96-well tissue culture plates and polymerized for 30 min at 37°C. Cells (1 × 10^4^/well) were trypsinized, resuspended in serum-free DMEM, and plated onto the top of the Matrigel. HUVECs (1.5 × 10^4^/well) seeded on the Matrigel bed and cultured in serum-free DMEM containing VEGF (0.3 nmol/L) were served as the positive control. Following incubation at 37°C for 3–24 h, each well was digitally photographed under a microscope (Leica) with phase contrast (magnification: 100x). The observed tubes and branching points were counted. Meanwhile, total tubular length was quantified by ImageJ software (National Institutes of Health, MA, USA) and calculated as the average of the total tubule length from three wells, three to five random fields per well.

### 2.8. Quantitative Real-Time PCR (qRT-PCR)

Total RNAs were prepared from tissues or cells using TRIzol reagent (Invitrogen, Grand Island, NY, USA) according to the manufacturer's instructions. While the quality of the RNA was evaluated using spectrophotometry and denaturing agarose gel electrophoresis, a 1 *μ*g aliquot of purified total RNA was reverse transcribed in a total volume of 20 *μ*l using a PrimeScript RT reagent kit (Bio-Rad Laboratories, Hercules, CA, USA). The specific primers used for qRT-PCR analysis were as follows: hJagged1, forward 5′-CCTGAAGGGGTGCGGTATAT-3′, reverse 5′-GGAGTTGACACCATCGATGC-3′ and h18S rRNA, forward 5′-CGGCTACCACATCCAAGGAA-3′, reverse 5′-CTGGAATTACCGCGGCT-3′. Each real-time PCR reaction had the following components: 1 *μ*L of RT product, 10 *μ*L of SYBR Green PCR Master Mix (Bio-Rad Laboratories), and 500 nM each of the forward and reverse primers. QRT-PCR was performed on a MyiQ Single Color Real-time PCR Detection System (Bio-Rad Laboratories) by the below procedure (95°C, 3 min, 94°C 10 s, 60°C 30 s, 72°C 30 s, 40 cycles). h18S rRNA was used as an internal control for Jagged1 detection. The samples were processed in duplicate using RNA preparations from 3 independent experiments. The fold change in Jagged1 expression was calculated using the 2^−ΔΔCT^ method.

### 2.9. Western Blot

The cells at passage 3 were rinsed twice with precooled PBS, then 1 mL of cell lysis buffer (50.0 mmol/L Tris pH = 7.6, 150.0 mmol/L NaCl, 0.1% SDS, 1.0% NP-40, protease inhibitor cocktail) was added, and the cells were scraped off. The cells were lysed at 4°C for 30 min under rotation and centrifuged at 15000 rpm for 30 min, and the supernatant was collected. Protein concentrations were determined by the BCA Protein Assay Reagent (Thermo Fisher Scientific, Rockford, IL, USA), after which 25 *μ*g of total proteins was loaded to 10% SDS-PAGE gel electrophoresis and transferred to a PVDF membrane (PVDF, Millipore) using the conventional method. The membrane was immunoblotted with primary antibodies against CD146 (1 : 500, ab75769, Abcam), Jagged1 (1 : 500, ab109536, Abcam), DLL4 (1 : 500, ab7280, Abcam), or GAPDH (1 : 10000, AP0063, Bioworld technology), followed by incubation with a goat anti-mouse (1 : 10000, BS12478, Bioworld technology) or goat anti-rabbit (1 : 10000, A0545, Sigma) secondary antibody. The bands were detected using an enhanced chemiluminescence kit (Amersham Biosciences Corp., Piscataway, NJ, USA), and densitometric analysis of each band was performed with Quantity-one (Bio-Rad) software.

### 2.10. Gene Microarray

Isolation and quality of total RNA were measured according to the above methods. Microarray analysis was used to screen changes in genome-wide gene expression patterns in UCA-PSCs, UCV-PSCs, and WJ-MSCs separated from the same human UC. The changes in 28264 human gene expression patterns were assessed by Phalanx Biotech gene microarray using the Human HOA7.1 One Array Plus (Phalanx Biotech Group, San Diego, CA).

### 2.11. Small Interfering RNA Transfection

Small interfering RNA (siRNA) was purchased from Ribo Life Science Co., Ltd. The three stem cells were transfected with Jagged 1 siRNA (si-Jagged1) (50 nM) or negative control siRNA (si-NC) (50 nM) in mediation of Lipofectamine™ 2000 Transfection Reagent (Invitrogen Inc., Carlsbad, CA, USA). Cells in each group were seeded in a 6-well plate and cultured in an incubator at 37°C with 5% CO_2_ until 80% confluence. Cell transfection was performed strictly according to the operation manual of Lipofectamine 2000 Transfection Reagent. The knockdown efficiency was confirmed at 48 h and 72 h posttransfection by RT-qPCR and western blot analysis, respectively. Then, in vitro angiogenic properties of the three stem cells were determined at 72 h after transfection.

### 2.12. Statistical Analysis

Each experiment was repeated at least 3 times. All values were expressed as the means ± standard error (SE). A two-tailed Student's *t*-test was used to evaluate the differences between two groups. The statistical significance of the difference among multiple comparisons was determined by one-way analysis of variance using Statistics Package for Social Science (SPSS 22.0, SPSS, Chicago, IL, USA). A *P* value <0.05 was considered statistically significant.

## 3. Results

### 3.1. Expression of PDGF-R*β*, NG2, *α*-SMA, and CD146 in Human UC

**Figure 1:**
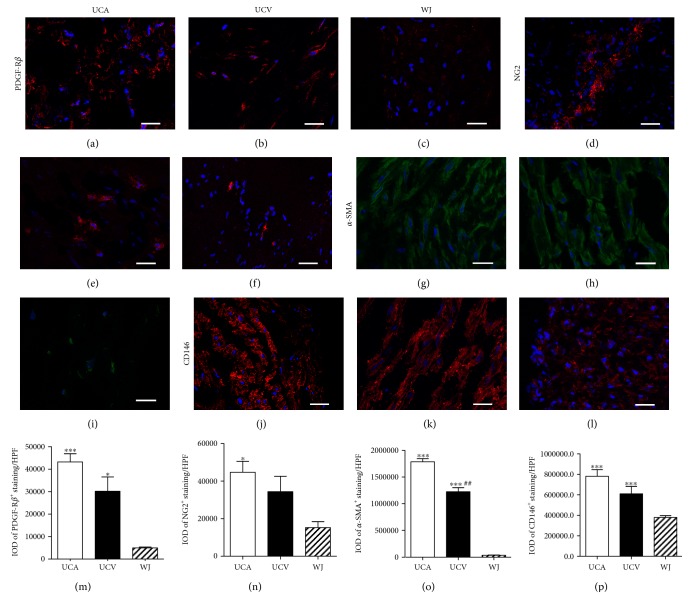
Immunolocalization of PDGF-R*β*, NG2, *α*-SMA, and CD146 in the human umbilical cord. Fluorescence imaging revealed a high incidence of PDGF-R*β*-positive (a–c), NG2-positive (d–f), *α*-SMA-positive (g–i), and CD146-positive (j–l) cells in the perivascular region. Bar: 25 *μ*m. The integrated optical density (IOD) values of positive staining in five randomly selected high power fields of view were counted. ^∗∗∗^*P* < 0.001, versus WJ-MSCs. ^∗^*P* < 0.05, versus WJ-MSCs. ^##^*P* < 0.01, UCV-PSCs versus UCA-PSCs.

PDGF-R*β* is a platelet-derived growth factor receptor which is involved in pericyte formation and recruitment during blood vessel morphogenesis. NG2 is a proteoglycan associated with pericytes during vascular morphogenesis. *α*-SMA can be reproducibly detected in cells surrounding the venules and arterioles and is responsible for regulating microvessel contractility. CD146 is an endothelial cell antigen expressed at the surface of pericytes [12]. In this study, immunofluorescence staining was used to visualize the expression of PDGF-R*β*, NG2, *α*-SMA, and CD146 in UCA, UCV, and WJ samples obtained from the same UC. The results revealed high expression of PDGF-R*β* in the perivascular region while PDGF-R*β*^+^ cells were scarcely detected in the WJ (Figures 1(a), 1(b), and 1(c)). NG2^+^ cells were primarily distributed in the UCA, followed by the UCV, while there was almost no NG2^+^ cells in WJ (Figures 1(d), 1(e), and 1(f)). *α*-SMA staining revealed a similar distribution pattern (Figures 1(g), 1(h), and 1(i)). These data demonstrated that pericyte markers (PDGF-R*β*, NG2, and *α*-SMA) were detected primarily in the perivascular region. CD146 expression was highly prevalent in the perivascular region, especially in the UCA (Figure 1(j)), followed by UCV (Figure 1(k)), but CD146 expression in the WJ was very low (Figure 1(l)). Quantitative analysis of the immunostaining showed that there were more PDGF-R*β*^+^ (Figure 1(m)), NG2^+^ (Figure 1(n)), *α*-SMA^+^ (Figure 1(o)), and CD146^+^ (Figure 1(p)) cells in the perivascular region than in WJ which suggested that the UCA and UCV walls contained most pericytes of UC.

### 3.2. Phenotypes of UCA-PSCs, UCV-PSCs, and WJ-MSCs

**Figure 2:**
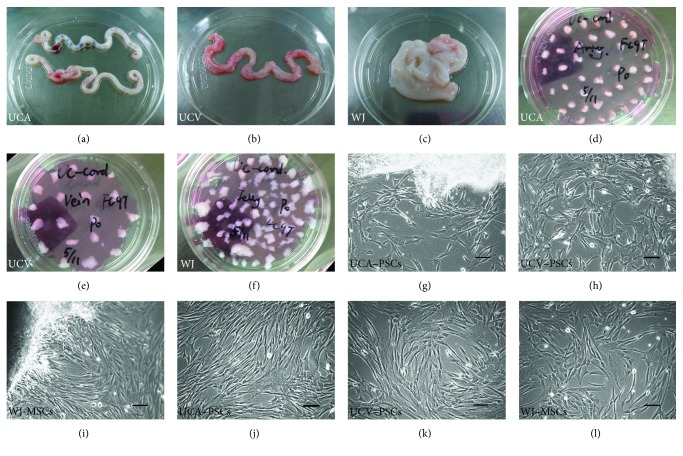
Isolation and characterization of umbilical cord artery perivascular stem cells (UCA-PSCs), umbilical cord vein perivascular stem cells (UCV-PSCs), and Wharton's jelly mesenchymal stem cells (WJ-MSCs). (a–c) Three different compartments in human umbilical cord: umbilical arteries (UCA) (a), umbilical vein (UCV) (b), and Wharton's jelly (WJ) (c). (d–f) Isolation of UCA-PSCs (d), UCV-PSCs (e), and WJ-MSCs in the human umbilical cord (f). (g–i) Cells from the third passage showed similar fibroblastic morphology. Bar: 100 *μ*m.

UCA-PSCs, UCV-PSCs, and WJ-MSCs were isolated from human UC using tissue explants. The obtained UCA, UCV, and WJ tissue samples were cut into small fragments and plated in dishes (Figures 2(a), 2(b), 2(c), 2(d), 2(e), and 2(f)). On days 7–10 after incubation, fibroblast-like cells migrated out of the tissues (Figures 2(g), 2(h), and 2(i)). The morphology of stem cells derived from the three different tissues was similar (Figures 2(j), 2(k), and 2(l)).

**Figure 3:**
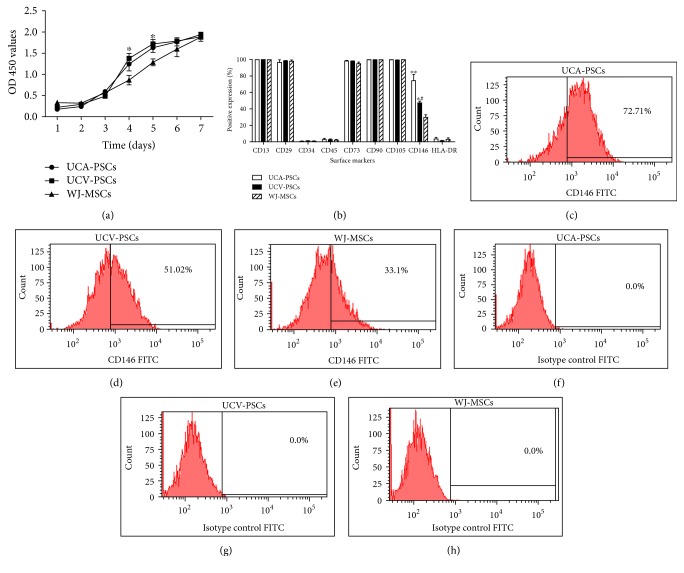
Proliferation and phenotype profile of umbilical cord artery perivascular stem cells (UCA-PSCs), umbilical cord vein perivascular stem cells (UCV-PSCs), and Wharton's jelly mesenchymal stem cells (WJ-MSCs). (a) Cell proliferation was continuously monitored for 7 days using cell-counting kit-8 (CCK-8), which showed that the number of UCA-PSCs was significantly higher than that of UCV-PSCs at days 4 and 5. However, the cell growth rate of UCA-PSCs, UCV-PSCs, and WJ-MSCs did not significantly differ. Bars represent the means ± SE of three independent experiments performed in triplicate. ^∗^*P* < 0.05, UCV-PSCs versus UCA-PSCs. (b) Profiles of cell surface epitopes in UCA-PSCs, UCV-PSCs, and WJ-MSCs. Abundance of cells positive for CD13, CD29, CD34, CD45, CD73, CD90, CD105, CD146, and HLA-DR, expressed as percentages, in UCA-PSCs, UCV-PSCs, and WJ-MSCs. Bars represent the means ± SE of donor samples (*n* = 3). ^∗∗^*P* < 0.01, UCA-PSCs versus WJ-MSCs. ^∗^*P* < 0.05, UCV-PSCs versus WJ-MSCs. ^#^*P* < 0.05, UCV-PSCs versus UCA-PSCs. (c–h) Representative flow cytometric plots, including isotype control (IgG-FITC) (f–h).

CCK-8 assays showed that UCA-PSCs, UCV-PSCs, and WJ-MSCs had similar proliferation tendency at passage 3 (Figure 3(a)). However, on days 4 and 5, UCA-PSCs had a significantly higher growth rate compared to UCV-PSCs.

Flow cytometric analysis of cells at passage 3 revealed that all these three cell populations were positive for CD13, CD29, CD73, CD90, and CD105 but negative for CD34, CD45, and HLA-DR (Figure 3(b)), which was consistent with the previous reports on MSC surface markers [1]. However, UCA-PSCs and UCV-PSCs had more CD146^+^ cells than WJ-MSCs (*P* < 0.05; Figures 3(b), 3(c), 3(d), 3(e), 3(f), 3(g), and 3(h)). CD146^+^ cells in many human tissues have been identified as MSC origin in vivo and have higher multilineage differentiation potential [22].

### 3.3. Multilineage Differentiation Potential of UCA-PSCs, UCV-PSCs, and WJ-MSCs

**Figure 4:**
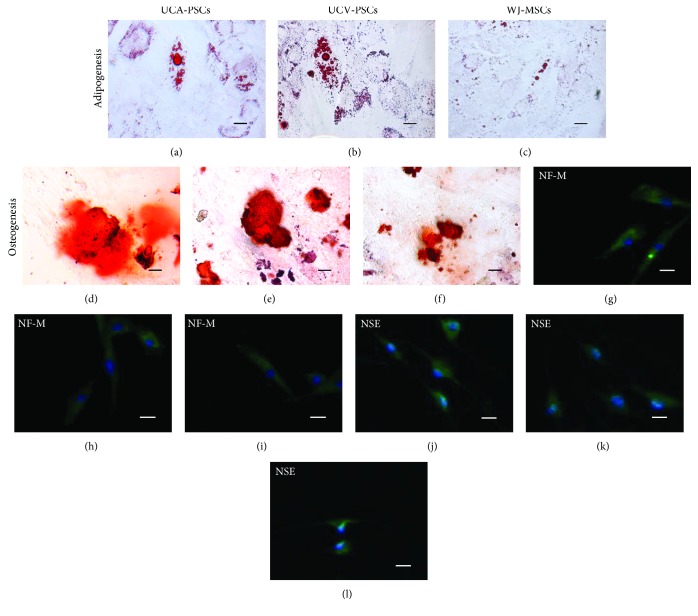
Differentiation of umbilical cord artery perivascular stem cells (UCA-PSCs), umbilical cord vein perivascular stem cells (UCV-PSCs), and Wharton's jelly mesenchymal stem cells (WJ-MSCs). (a–c) For adipogenic differentiation, the cells were cultured in adipogenic induction medium for 14 days. The formation of lipid droplets was confirmed by oil red O staining. (d–f) Cells were cultured in osteogenesis induction medium for 21 days. Calcium deposition was confirmed by alizarin red staining. (g–l) Differentiation of cells to neuronal lineage after 18 h preinduction and 36 h induction was confirmed by immunofluorescence staining of neurofilament medium polypeptide (g–i) and neuron-specific enolase (j–l). Bar: 10 *μ*m.

To study whether the MSCs derived from perivascular regions and WJ had similar multilineage differentiation capacity, cells from passage 3 were cultured under various conditions for adipogenic, osteogenic, and neural-like differentiation. For adipogenic differentiation, lipid-containing cells were detected earlier in UCA-PSCs and UCV-PSCs than in WJ-MSCs (day 10, day 10, and day 12, resp.; data not shown). At 14 days after induction, the three stem cell populations were all capable of differentiating into adipocytes containing lipid droplets (Figures 4(a), 4(b), and 4(c)). For osteogenesis, bone nodules were first detected in UCA-PSCs and UCV-PSCs but not in WJ-MSCs after the cell populations were cultured under osteogenic conditions on day 10 (data not shown). Three weeks later, alizarin red S staining revealed a greater extent of mineralization with detectable bone nodules in all three stem cell populations (Figures 4(d), 4(e), and 4(f)). The neural-like differentiation of the stem cells was confirmed by NF-M (Figures 4(g), 4(h), and 4(i)) and NSE (Figures 4(j), 4(k), and 4(l)) using immunofluorescence staining. There were no differences in neural differentiation capacity among the three MSCs. These results suggested that UCA-PSCs, UCV-PSCs, and WJ-MSCs all had multilineage differentiation potential, but UCA-PSCs and UCV-PSCs clearly had a higher ability toward mesoderm lineage differentiation.

### 3.4. UCA-PSCs Exhibited Better Angiogenesis Capacity In Vitro

**Figure 5:**
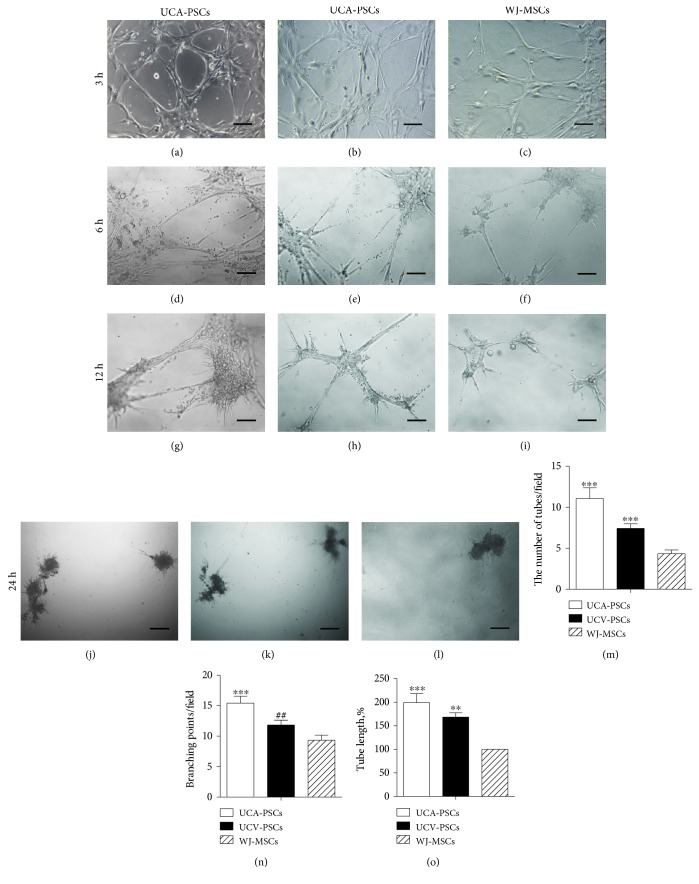
In vitro Matrigel tube formation assay. Umbilical cord artery perivascular stem cells (UCA-PSCs), umbilical cord vein perivascular stem cells (UCV-PSCs), and Wharton's jelly mesenchymal stem cells (WJ-MSCs). Cells (1 × 10^4^/well) were treated with a low concentration of glucose and plated on Matrigel in 96-well tissue culture plates. Tube formation was microscopically compared after 3 h (a–c), 6 h (d–f), 12 h (g–i), and 24 h (j–l). Bar: 60 *μ*m. The number of tubes (m) and branching point per field (n) and the total length of tubes per field (o) were quantified 3 h after treatment by counting 3–5 random fields/well under the microscope (magnification: 100x). UCA-PSCs and UCV-PSCs, especially UCA-PSCs, displayed greater tube formation ability than WJ-MSCs. *n* = 5. ^∗∗∗^*P* < 0.001, versus WJ-MSCs. ^∗∗^*P* < 0.01, UCV-PSCs versus WJ-MSCs. ^##^*P* < 0.01, UCV-PSCs versus UCA-PSCs.

To compare angiogenesis capacity between UCA-PSCs, UCV-PSCs, and WJ-MSCs, tube formation assays were carried out to investigate the capacity of differentiation into a capillary-like structure. As shown in Figures 5(a), 5(b), and 5(c), UCA-PSCs, UCV-PSCs, and WJ-MSCs were cultured on Matrigel-coated plates for 3 h. Microscopic observation revealed that the number of tubules per random field was apparently higher in UCA-PSCs and UCV-PSCs than in WJ-MSCs. At 6 h, the tubes had partly disintegrated in all three cell populations (Figures 5(d), 5(e), and 5(f)). Meanwhile, tube formation in HUVECs was observed as positive control (Supplemental Figure 1() available online at https://doi.org/10.1155/2017/3175748). Interestingly, tube-like structures remained in UCA-PSCs even after 12 h on Matrigel but disappeared in UCV-PSCs and WJ-MSCs (Figures 5(g), 5(h), and 5(i)), indicating that UCA-PSCs had advantages over UCV-PSCs and WJ-MSCs in maintaining the stability of the formed tubes. At 24 h, all the tube-like structures degraded in these three stem cell populations (Figures 5(j), 5(k), and 5(l)). Statistical analysis showed that the number of tubes per field significantly increased in UCA-PSCs (11.08 ± 1.29) and UCV-PSCs (7.42 ± 0.57) compared to those in WJ-MSCs (4.33 ± 0.47; *P* < 0.001; Figure 5(m)). In addition, the number of tube branch points per field was higher in UCA-PSCs (15.42 ± 1.14) and UCV-PSCs (11.83 ± 0.79) than in WJ-MSCs (9.33 ± 2.87; *P* < 0.001, UCA-PSCs versus WJ-MSCs; Figure 5(n)). Meanwhile, the total tube length was significantly longer in UCA-PSCs (199.27% ± 18.90%; *P* < 0.001; Figure 5(o)) and UCV-PSCs (168.53% ± 9.09%; *P* < 0.01; Figure 5(o)) compared to that in WJ-MSCs. Moreover, the number of tube branch points per field was significantly higher in UCA-PSCs than in UCV-PSCs (*P* < 0.01), while there were no significant differences in the number and length of tubes between UCA-PSCs and UCV-PSCs (*P* > 0.05). These results demonstrated that UCA-PSCs and UCV-PSCs, especially UCA-PSCs, exhibited better angiogenic ability compared to the WJ-MSCs in vitro.

### 3.5. Higher Expression of CD146 and Jagged1 in UCA-PSCs

**Figure 6:**
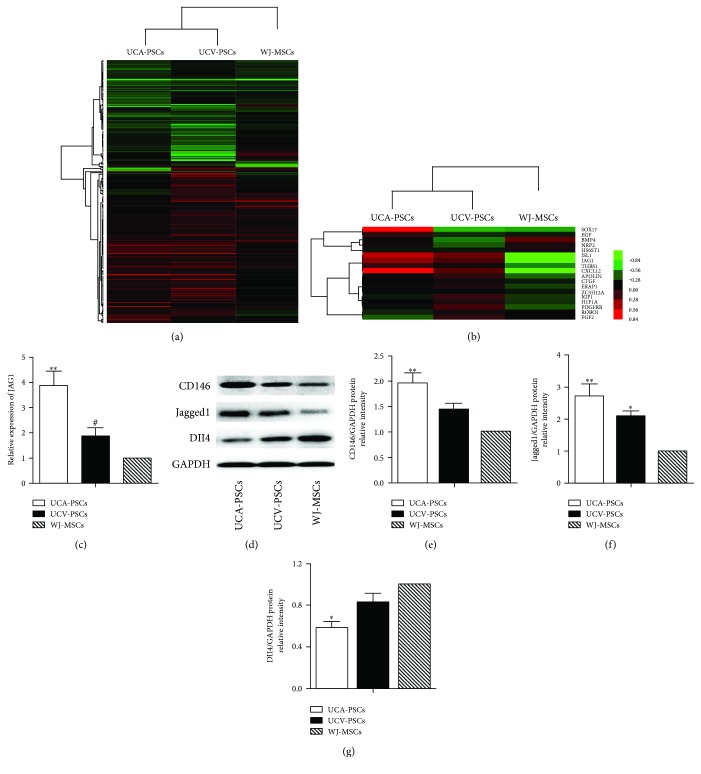
Expression of angiogenesis-related genes in the umbilical cord artery perivascular stem cells (UCA-PSCs), umbilical cord vein perivascular stem cells (UCV-PSCs), and Wharton's jelly mesenchymal stem cells (WJ-MSCs). (a) Comparison of gene expression patterns in UCA-PSCs, UCV-PSCs, and WJ-MSCs using microarray analysis. Red indicates upregulated genes while green indicates downregulated genes. (b) Gene expression heatmaps showed fold changes in the expression of a selection of genes involved in angiogenesis. (c) mRNA levels of Jagged1 in UCA-PSCs, UCV-PSCs, and WJ-MSCs measured by qRT-PCR. ^∗∗^*P* < 0.01, UCA-PSCs versus WJ-MSCs. ^#^*P* < 0.05, UCV-PSCs versus UCA-PSCs. (d) Western blot analysis revealed that the CD146 and Jagged1 expression levels were the highest in UCA-PSCs, followed by UCV-PSCs and WJ-MSCs. While the Delta-like ligand (Dll4) protein displayed the opposite expression pattern in these three cell types. Quantitative analysis of CD146 (e), Jagged1 (f), and Dll4 (g) protein expression levels in UCA-PSCs, UCV-PSCs, and WJ-MSCs. Data are representative of three independent experiments performed in triplicate, and the expression of targeted protein was relative to the expression of GAPDH protein. ^∗∗^*P* < 0.01, versus WJ-MSCs. ^#^*P* < 0.01, UCV-PSCs versus UCA-PSCs. ^∗^*P* < 0.05, versus WJ-MSCs.

We carried out a genome-wide gene profile analysis to further investigate the biological characteristics of UCA-PSCs, UCV-PSCs, and WJ-MSCs. As shown in the clustering analysis, based on 293 selected differentially expressed genes, UCA-PSCs were more closely related to UCV-PSCs than to WJ-MSCs (Figure 6(a)). In addition, many angiogenesis-related genes, such as *ISL1*, *JAG1*, *THBS1*, *CXCL12*, *CTGF*, *HIF1A*, and *ERAP1*, were increased in UCA-PSCs and UCV-PSCs than in WJ-MSCs (Figure 6(b)). Furthermore, Jagged1 expression was the highest in UCA-PSCs, followed by UCV-PSCs, and then WJ-MSCs (Figure 6(b)). Jagged1 is well-known to play an important role in both physiological and pathological angiogenesis [23]. The Jagged1 mRNA levels were measured by qRT-PCR, and the results were in line with those of the microarray analysis (Figure 6(c)). The results of the western blot analysis also confirmed that UCA-PSCs had the highest CD146 and Jagged1 protein expression, followed by UCV-PSCs and WJ-MSCs. However, the protein level of Delta-like ligand (Dll4), another important ligand in Notch signals [24], revealed the opposite expression pattern among the three cell populations, which may be beneficial in angiogenesis (Figure 6(d)). Statistical analysis of the different protein levels of CD146 (Figure 6(e)), Jagged1 (Figure 6(f)), and Dll4 (Figure 6(g)) in UCA-PSCs, UCV-PSCs, and WJ-MSCs was also showed.

### 3.6. Knockdown of Jagged1 Decreased Tubule Formation in UCA-PSCs

**Figure 7:**
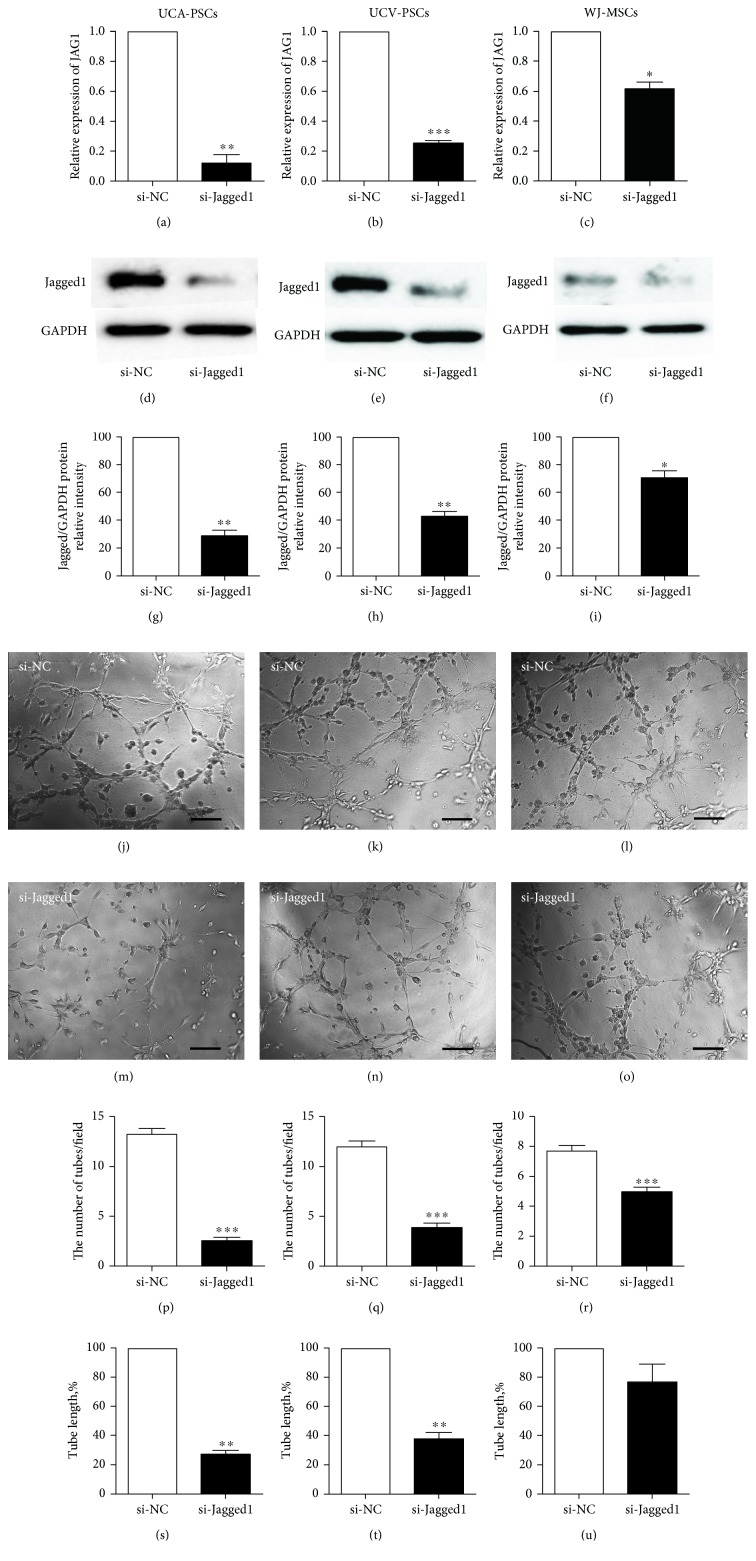
Knockdown of Jagged1 decreased tubule-like structures formation in the umbilical cord artery perivascular stem cells (UCA-PSCs), umbilical cord vein perivascular stem cells (UCV-PSCs), and Wharton's jelly mesenchymal stem cells (WJ-MSCs). (a–i) Jagged1 knockdown was effective. qRT-PCR analysis of Jagged1 mRNA levels in UCA-PSCs (a), UCV-PSCs (b), and WJ-MSCs (c) 48 h after the transfection with negative control siRNA (si-NC) or Jagged1 siRNA (si-Jagged1). ^∗∗^*P* < 0.01, si-Jagged1 versus si-NC; ^∗∗∗^*P* < 0.001, si-Jagged1 versus si-NC; ^∗^*P* < 0.05, si-Jagged1 versus si-NC. Western blot analysis of Jagged1 expression in UCA-PSCs (d), UCV-PSCs (e), and WJ-MSCs (f) 72 h after the transfection with si-NC or si-Jagged1. Quantitative analysis of Jagged1 protein expression levels in UCA-PSCs (g), UCV-PSCs (h), and WJ-MSCs (i) 72 h after the transfection with si-NC or si-Jagged1. Data are representative of three independent experiments performed in triplicate, and the expression of targeted protein was relative to the expression of GAPDH protein. ^∗∗^*P* < 0.01, si-Jagged1 versus si-NC; ^∗^*P* < 0.05, si-Jagged1 versus si-NC. Knockdown of Jagged1 decreased angiogenesis in UCA-PSCs, UCV-PSCs, and WJ-MSCs in vitro. (j–o) Representative images of tubular structures. Bar: 50 *μ*m. Tube formation assays were performed 72 h after transfection of si-NC or si-Jagged1. The number of tubes in UCA-PSCs (p), UCV-PSCs (q), and WJ-MSCs (r), together with the total length of tubes per field in UCA-PSCs (s), UCV-PSCs (t), and WJ-MSCs (u) were quantified 3 h after treatment by counting 3–5 random fields/well under the microscope (magnification: 100x). *n* = 5. ^∗∗∗^*P* < 0.001, si-Jagged1 versus si-NC; ^∗∗^*P* < 0.01, si-Jagged1 versus si-NC.

To examine the role of Jagged1 in angiogenesis, in particular the role of endogenous Jagged1 in capillary tube formation of the three stem cells, siRNA was used to silence the expression of Jagged1 in UCA-PSCs, UCV-PSCs, and WJ-MSCs. The results showed that the depletion of Jagged1 in UCA-PSCs, UCV-PSCs, and WJ-MSCs decreased Jagged1 mRNA level compared with the si-NC group (Figures 7(a), 7(b), and 7(c)). Similarly, the three stem cells transfected with si-Jagged1 expressed the lower protein level of Jagged1 at 72 hours after transfection (Figures 7(d), 7(e), and 7(f)). Transfection of si-Jagged1 resulted in a 71.26%, 57.38%, and 29.51% decrease of Jagged1 expression in UCA-PSCs (Figure 7(g)), UCV-PSCs (Figure 7(h)), and WJ-MSCs, respectively (Figure 7(i)). Then, we determined the effect of Jagged1 knockdown on the angiogenic properties of the three stem cells in vitro. As shown in Figures 7(j), 7(k), 7(l), 7(m), 7(n), and 7(o), UCA-PSCs, UCV-PSCs, and WJ-MSCs transfected with si-Jagged1 or si-NC were cultured on Matrigel-coated plates for 3 h. Representative images revealed that the number of tubule-like structures per random field was apparently lower in si-Jagged1 group, compared with si-NC group in UCA-PSCs (Figures 7(j) and 7(m)), UCV-PSCs (Figures 7(k) and 7(n)), and WJ-MSCs (Figures 7(l) and 7(o)). Statistical analysis showed that Jagged1 knockdown led to a significant reduction in the number of tubes per field in UCA-PSCs (2.56 ± 0.33 versus 13.22 ± 0.67; *P* < 0.001, si-Jagged1 versus si-NC; Figure 7(p)), UCV-PSCs (3.85 ± 0.45 versus 11.96 ± 0.56; *P* < 0.001, si-Jagged1 versus si-NC; Figure 7(q)) and WJ-MSCs (4.96 ± 0.30 versus 7.70 ± 0.32; *P* < 0.001, si-Jagged1 versus si-NC; Figure 7(r)). In addition, compared to the si-NC group, the total tube length of si-Jagged1 group decreased by 72.74% in UCA-PSCs (*P* < 0.01; Figure 7(s)), 62.27% in UCV-PSCs (*P* < 0.01; Figure 7(t)); and 23.38% in WJ-MSCs (*P* > 0.05; Figure 7(u)). These data suggested that Jagged1 played a vital role in tube formation in the three stem cells.

## 4. Discussion

Human UC-derived mesenchymal stem cells are a promising versatile tool for regenerative medicine and immunotherapy [25]. This is the first study to compare the features of UCA-PSCs, UCV-PSCs, and WJ-MSCs obtained from the same human UC. Our results revealed that UCA-PSCs expressed higher levels of CD146 than WJ-MSCs. Additionally, UCA-PSCs and UCV-PSCs, especially UCA-PSCs, showed greater angiogenesis capacity and expressed higher levels of Jagged1, which is an important Notch ligand in angiogenesis. Our study demonstrated that the knockdown of Jagged1 decreased tubule-like structure formation in UCA-PSCs, UCV-PSCs, and WJ-MSCs.

As a prerequisite to the identification of human perivascular cells, we used immunofluorescence assay to detect relevant marker combinations for this elusive cell population. It has been confirmed that all perivascular cells (pericytes) still display overextended culture, the markers their ancestors natively expressed in the tissue of origin (PDGF-R*β*, NG2, *α*-SMA, and CD146) [26]. In the present study, pericyte markers were detected in the perivascular region and the UCA had a higher proportion of cells expressing CD146 and NG2, indicating that there may be more perivascular stem cells surrounding UCA.

Meanwhile, the expression of CD146 was most notably elevated in UCA-PSCs and UCV-PSCs compared to WJ-MSCs, which was indicated not only by flow cytometry analysis of the harvested cell populations from the perivascular region but also by direct immunostaining of the human UC samples. CD146 is identified as a potential marker for multipotency [27]. The CD146^+^ subset of MSCs can long-time maintain the hematopoietic stem cells (HSCs) with engraftment and self-renewal ability [28]. In an experimental approach combining stringent cell purification by flow cytometry and differentiation in culture and in vivo, human CD146^+^ perivascular cells represent the ubiquitous ancestors of MSCs [29]. Additionally, CD146 has been reported to play a crucial role in the vascular development [30]. A previous study found that knockdown of CD146 protein expression severely hindered vascular development, leading to poorly developed intersomitic vessels, with lack of blood flow through the intersomitic vessel region [31]. In addition, the gain-of-function analysis of CD146 in zebrafish found that enforcing expression of CD146 induced sprouting angiogenesis [32]. Moreover, as a novel VEGFR-2 coreceptor, CD146 is required in the promotion of endothelial cell migration and microvascular formation [33].

A complex labyrinth of blood vessels in the human body provides cells and tissues with the nutrients and oxygen needed for survival, proliferation, and a variety of physiological activities. The majority of the blood vessel network is considered to be built through angiogenic processes. Normal physiological angiogenesis is the formation of new blood vessels from preexisting vasculature, and it is a fundamental event during embryonic development, homeostasis, wound and fracture healing, and the growth and function of the female reproductive organs [34–36]. Thus, understanding the angiogenesis ability of these three MSC populations is vital in consideration of their clinical application. In the present study, we confirmed that UCA-PSCs had better tube formation capacity in vitro than WJ-MSCs. In addition to the increased number of tubes, branching points and total tube length per field, UCA-PSCs and UCV-PSCs, particularly UCA-PSCs, were also superior to WJ-MSCs in maintaining the stability of the tubes. Perivasculature has been considered to be the niche for various types of MSCs [37]. However, whether arteries, veins, and capillaries represent different MSC niches remains largely unknown. A recent study suggested that mouse incisor MSCs were localized around arterioles alone and not veins or capillaries and were regulated by the neurovascular bundle niche [38]. This finding may give a possible explanation as to why UCA-PSCs had a slight advantage over UCV-PSCs in terms of angiogenic capacity.

In addition to CD146, UCA-PSCs and UCV-PSCs both expressed higher levels of angiogenesis-related genes than WJ-MSCs, such as *ISL1*, *JAG1*, *THBS1*, *CXCL12*, *CTGF*, *HIF1A*, and *ERAP1*. It was reported that Jagged1 overexpression in tumor cells enhances neovascularization and tumor growth and that loss of Jagged1 in endothelial had an inhibitory effect on the neoangiogenic and maturation responses as well as an angiocrine effect in tumor cells [39]. Furthermore, mutations in the human Jagged1 gene cause Alagille syndrome, which involves complex cardiac defects and vascular anomalies [40]. Our data showed that knockdown of Jagged1 expression by siRNA in UCA-PSCs, UCV-PSCs, and WJ-MSCs resulted in remarkably reduced tube formation in vitro. However, the knockdown efficiency in WJ-MSCs was lower compared to the other two kinds of cell, which may be explained by the low expression of endogenous Jagged1. During the process of angiogenesis, a well-regulated balance between the migration of tip cells and proliferation of stalk cells is essential for adequately shaped nascent sprouts [41]. Selecting the tip and the stalk fate is critical for developing a functional vessel and mediated by the Notch signaling pathway, a conserved cell-cell communication pathway activated through transinteractions between Notch ligands and receptors [42]. The Notch ligands Jagged1 and Dll4 have opposing effects on angiogenesis. Different signals might modulate angiogenesis by changing the ratio of Jagged1 and Dll4 expression, which integrated pro- or antiangiogenic signal into the selection of endothelial tip cells [43], which may be the cause of optimum angiogenic capacity of UCA-PSCs.

## 5. Conclusions

In summary, our results indicated for the first time that UCA-PSCs and UCV-PSCs, especially UCA-PSCs, had better angiogenesis capacity than WJ-MSCs in vitro. In addition, higher expression level of angiogenesis related genes, such as CD146 and Jagged1, was detected in UCA-PSCs. These results offered a promising candidate, UCA-PSCs, for cell-based therapy for ischemia.

## Supplementary Material

Supplemental Figure 1. In vitro matrigel tube formation assay of HUVECs at 6 h. HUVECs (1.5 × 10^4/well) were plated on Matrigel and cultured in serum-free DMEM containing VEGF (0.3 nmol/L). Tube formation was microscopically observed after 6 h. Bar: 60 μm.
